# The role of mpMRI in qualification of patients with ISUP 1 prostate cancer on biopsy to radical prostatectomy

**DOI:** 10.1186/s12894-021-00850-3

**Published:** 2021-05-18

**Authors:** Łukasz Nyk, Omar Tayara, Tomasz Ząbkowski, Piotr Kryst, Aneta Andrychowicz, Wojciech Malewski

**Affiliations:** 1 Second Department of Urology, Centre of Postgraduate Medical Education, Warsaw, Poland; 2 Department of Urology, Military Institute of Medicine, Warsaw, Poland; 3 Urological Clinic, Warsaw, Poland

**Keywords:** Active surveillance, Gleason upgrading, Prostate cancer, MpMRI of prostate, PIRADS score

## Abstract

**Background:**

To investigate the role of mpMRI and high PIRADS score as independent triggers in the qualification of patients with ISUP 1 prostate cancer on biopsy to radical prostatectomy.

**Methods:**

Between January 2017 and June 2019, 494 laparoscopic radical prostatectomies were performed in our institution, including 203 patients (41.1%) with ISUP 1 cT1c-2c PCa on biopsy. Data regarding biopsy results, digital rectal examination, PSA, mpMRI and postoperative pathological report have been retrospectively analysed.

**Results:**

In 183 cases (90.1%) mpMRI has been performed at least 6 weeks after biopsy. Final pathology revealed ISUP Gleason Grade Group upgrade in 62.6% of cases. PIRADS 5, PIRADS 4 and PIRADS 3 were associated with Gleason Grade Group upgrade in 70.5%, 62.8%, 48.3% of patients on final pathology, respectively. Within PIRADS 5 group, the number of upgraded cases was statistically significant.

**Conclusions:**

PIRADS score correlates with an upgrade on final pathology and may justify shared decision of radical treatment in patients unwilling to repeated biopsies. However, the use of PIRADS 5 score as a sole indicator for prostatectomy may result in nonnegligible overtreatment rate.

## Background

Prostate cancer is diagnosed in 1.1 million men annually [1]. The introduction of prostate specific antigen (PSA) screening attributes to common low risk disease diagnoses [2]. The indolent nature of the majority of cases encourages preservative management of this type of malignancy. Active surveillance (AS) is a recommended approach in low risk prostate cancer and selected cases of intermediate disease [3]. It aims to avoid serious complications of radical treatments such as incontinence or erectile dysfunction. AS studies report high cancer specific survival rates of up to 99% at 15 years [4]. Despite promising outcomes, this management method still raises doubts as to proper selection criteria. There are several AS protocols recommending specific measures to include patients’ safely. Most of them are based on PSA and biopsy results as well as digital rectal examination (DRE). In recent years, intriguing diagnostic tool—multiparametric resonance imaging (mpMRI) of the prostate has been developed. The sensitivity in detecting clinically significant cancer regarding the Gleason grade group is 95% [5]. Nevertheless, mpMRI is not recommended in any protocol as selection criterion of AS. Several studies proved that among patients eligible for AS, upstaging and upgrading in postoperative pathology report accounts for up to 47.3% and 59.7% of cases, respectively [6]. The EAU Guidelines recommend mpMRI performance before the confirmatory biopsy within AS management, which should be repeated at least every 2–3 years [7]. However, multiple repeated biopsies are associated with an increase of inflammation within the prostate and may influence the course of potential prostatectomy procedure and its functional results [8, 9]. Moreover, prostate biopsy itself is not devoid of acute and chronic complications, some of which require hospitalization. Chronic consequences of prostate biopsy may involve even erectile dysfunction [10]. Given the efficacy of mpMRI in detecting clinically significant prostate cancer and disadvantages of repeated prostate biopsies, we hypothesized that some patients with ISUP Gleason Grade Group 1 (GS6) on biopsy and high prostate imaging and report and data system (PIRADS)score on confirmatory biopsy may be skipped and radical treatment can be offered.

This study aimed to investigate the role of mpMRI and high PIRADS score as independent triggers in the qualification of patients with ISUP 1 prostate cancer on biopsy to radical prostatectomy.

## Methods

Between January 2017 and June 2019, 494 laparoscopic radical prostatectomies with or without extended lymph node dissection were performed in our institution. Data regarding biopsy results, DRE, PSA, mpMRI, and postoperative pathological report have been prospectively summarized. We identified 203 patients with ISUP Gleason Grade Group 1(ISUP 1) on biopsy. Based on DRE and PSA level, included patients were subdivided into risk groups: low risk group (PSA < 10 ng/mL and cT < 1–2a), intermediate risk group (PSA 10–20 ng/mL or cT2b), and high risk group (PSA > 20 or cT2c) [11]. In 183 cases, mpMRI has been performed at least 6 weeks after biopsy. Every mpMRI have been assessed during qualification appointment and clinical rounds. The assessment was based on MR prostate imaging reporting and data system version 2.0, and proper PIRADS grades from 2 to 5 were ascribed to visible lesions. Within this group, no cases were suspected of extraprostatic extension (EPE) based on DRE. All patients who underwent laparoscopic radical prostatectomies due to ISUP 1 prostate cancer had thorough preoperative discussion about possible risk of overtreatment, complications, and other management options including AS.

All patients signed the informed consent that their medical data might be used in the future as part of retrospective study. The analyzed datasets were anonymised before we used it for the purpose of current study. According to the Act of 6 September 2001 Pharmaceutical Law the study was qualified as noninterventional observation study. Thus, this study did not require approval by Ethics Committee and registration in Central Register of Clinical Studies.

### Statistical analysis

Statistical analyses were performed using IBM SPSS Statistics 26.

The χ^2^ was used for the categorical and nonparametric continuous variable comparison to check a statistical significance. There were three significance levels determined namely: *p* < 0.001 (***), *p* < 0.01 (**), and *p* < 0.05 (*). This test was applied in the UPGRADE and GLS LPR comparison and showed a statistical significance. Furthermore, a correlation between GLS LPR and MRI PIRADS was analyzed and showed a correlation close to the statistical significance.

The Kruskal–Wallis test was used for the ordinal and measurement variable comparison to check for a statistical significance. There were three significance levels determined as *p* < 0.001 (***), *p* < 0.01 (**), and *p* < 0.05 (*). This test compared a correlation between PSA and GLS LPR as well as PSA and MRI PIRADS and showed statistically significant differences between PSA and GLS LPR variables; however, no statistical significance within PSA and MRI PIRADS variables.

In addition, the Mann‐Whitney U test was used for the MRI PIRADS and GLS LPR variable comparison. This test showed a statistical significance between the two groups. Categorical variables were listed using the number, and percentage values; however, nonparametric continuous variables were defined using the median, minimum, and maximum values. The odds ratio (OR) with 95% confidence interval (CI) were reported for each factor.

## Results

The analysis involved 183 patients with ISUP 1 on prostate biopsy with preoperative mpMRI. Median PSA was 9.4 ng/mL with a density of 0.20. According to DRE, there was no EPE suspicion in any case. MpMRI of the prostate indicated EPE suspicion in 5.6% of patients. Final pathological report revealed EPE in 13% of cases. According to EAU risk group classification, 88.7% of patients presented low and intermediate risk disease. According to PIRADS classification, PIRADS 5, 4, 3, and 2 lesions occurred in 32.8%, 47%, 16.4%, and 3.8%, respectively (Table 1). Distribution of variables: clinical stage according to DRE and mpMRI as well as corresponding pathological stage are presented in Table 1. Final pathology revealed ISUP Gleason Grade Group upgrade in 62.6% of cases.

**Table 1:** Characteristics of the study group

	Number of patients	% of patients
*Clinical stage based on DRE*		
cT1	40	19.7
cT2a	40	19.7
cT2b	119	58.6
cT2c	4	2
*Clinical stage based on MRI*		
iT0	7	3.4
iT2a	35	17.2
iT2b	40	19.7
iT2c	86	42.4
iT3a	10	4.9
iT3b	5	2.5
Missing	20	9.9
*PIRADS category in MRI*		
PIRADS 3	29	15,8
PIRADS 4	86	47
PIRADS 5	61	33,3
*BCR risk according to EAU criteria*		
Low	29	14.3
Intermediate	151	74.4
High	23	11.3

*BCR* biochemical recurrence, *DRE* digital rectal examination, *MRI* magnetic resonance imaging

ISUP Gleason Grade Group upgrade occurred in 126 of 203 patients (62.1%).

In the low risk group, there was no statistically significant difference in number of upgraded and non-upgraded cases on final pathological result (*p* < 0.098). The intermediate risk group entailed upgrade in 66.7% of cases.

**Fig. 1:**
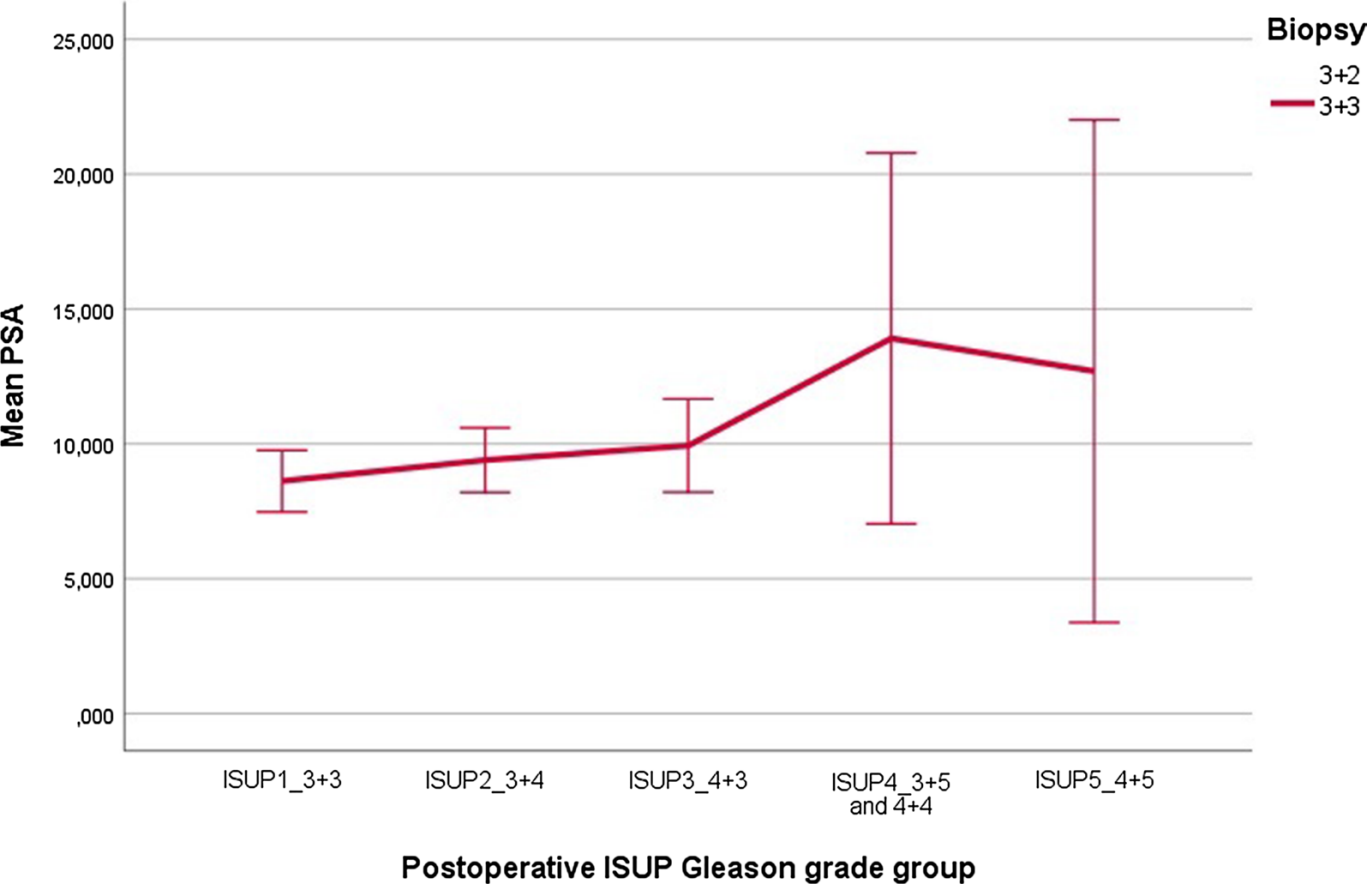
Postoperative ISUP Gleason grade group with regard to mean PSA

Moreover, there was statistically significant linear correlation between the PSA result and upgrade on final pathological result (Fig. 1).

**Fig. 2:**
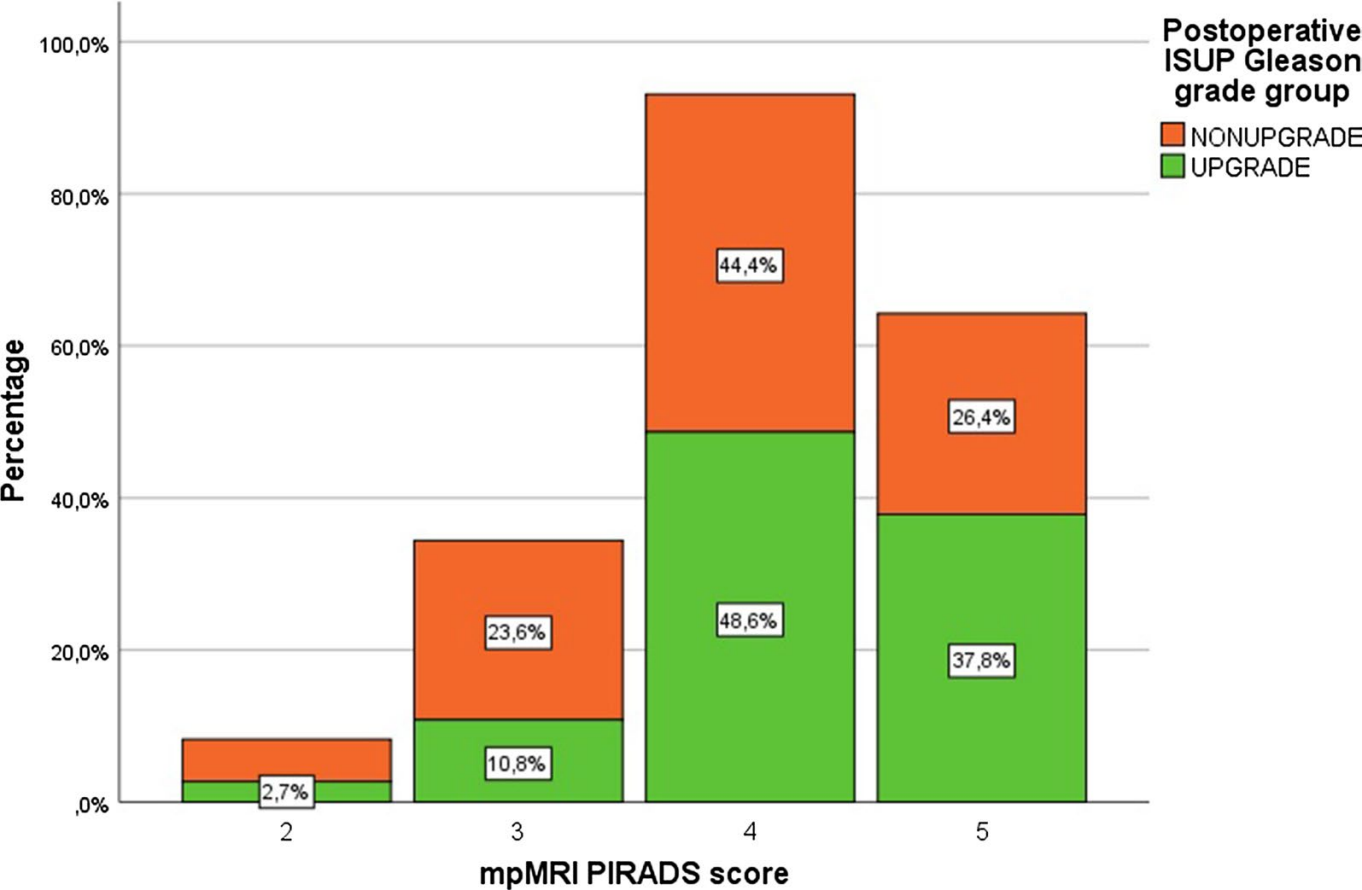
Postoperative ISUP Gleason grade group with regard to mpMRI PIRADS score

PIRADS 5, PIRADS 4 and PIRADS 3 were associated with Gleason Grade Group upgrade in 70.5%, 62.8%, 48.3% of patients on final pathology, respectively. Only within PIRADS 5 group, the difference between the number of upgraded and non-upgraded cases was statistically significant (*p* < 0.016) (Table 2, Fig. 2).

**Table 2:** Comparison of upgraded or nonupgraded cases within different PIRADS score groups

	Upgrade	Non-upgrade	Total n(%)	*p* value
ISUP1 + PIRADS5	43 (70.5%)	18 (29.5%)	61 (100%)	0.016
ISUP1 + PIRADS4	54 (62.7%)	32 (37%)	86 (100%)	0.062
ISUP1 + PIRADS3	12 (44%)	15 (55%)	27 (100%)	0.086

## Discussion

Management of low risk prostate cancer remains a controversial issue. Concerns about complications of the radical treatment encourage implementation of the conservative approach. Low risk prostate cancer is invariably associated with pathological diagnosis of ISUP Gleason Grade Group 1 (Gleason 6[3 + 3]) (ISUP 1) on prostate biopsy [12]. ISUP 1 prostate cancer lacks metastatic potential. There was no single lymph node metastasis among more than 14,000 prostate and lymph node specimens after radical treatment with ISUP 1 analyzed by Ross et al. However, cases with even a single microfocus of ISUP 1defined as 5% or less in one biopsy core might be upgraded and have adverse pathological features in up to 22% of patients [13]. Moreover, the large analysis of Surveillance, Epidemiology, and End Results database revealed that 44% of low risk prostate cancer defined as cT1c/2a, ISUP 1, and PSA below 10 ng/mL was upgraded at final pathological report [14]. Consequently, ISUP Gleason Grade Group 1 detection may indicate disease of metastatic potential in almost half of cases. Nevertheless, current guidelines recommend AS in any case of low risk prostate cancer and selected cases of intermediate risk disease. MpMRI of the prostate emerges as new diagnostic tool that is suggested to be used even before prostate biopsy. It is strongly recommended before second biopsy in case of persistent prostate cancer suspicion and before confirmatory biopsy within the AS protocol [15]. Standardization of mpMRI interpretation was first proposed within the PIRADS in 2012 [16, 17]. This protocol was improved in PIRADS version 2.0, where prostate lesions were scored from 1 to 5, and scores 4 and 5 indicate likely and very likely presence of significant cancer, respectively [18].

In our study, patients with ISUP 1 PCA on prostate biopsy who underwent preoperative mpMRI and subsequent laparoscopic radical prostatectomies were retrospectively analyzed. We assessed the correlation between mpMRI and final pathological results. To the best of our knowledge, upgrade is analyzed for the first time in terms of final pathological result after radical prostatectomy. The detection rate of clinically significant PCA is high and reaches up to 77% and 83% in case of PIRADS 5 score [19, 20].

Therefore, we hypothesized, that patients diagnosed with ISUP 1 prostate cancer and high PIRADS score confirmatory biopsy may be spared due to substantial probability of at least ISUP 2 prostate cancer in the final specimen.

Most modern studies evaluate the safety of avoidance of biopsy in case of negative mpMRI. They compare the mpMRI result with the biopsy specimen and indicate that the use of mpMRI may significantly decrease unnecessary biopsies [21]. Interestingly, in comparison to systematic biopsy, mpMRI fusion confirmatory biopsy resulted in nonsignificant upgrade rates [22].

On the other hand, Verep et al. looked for upgrading predictors by means of comparing the biopsy result and the final specimen. In this study, upgrade occurred in approximately half of all cases. PSA and PSA density were found to be the strongest upgrade predictors. However, no data on the correlation of upgrade with mpMRI score has been included [23].

MpMRI PIRADS 4 and 5 in the study presented by Propilia et al. were associated with the upgrade of ISUP score in 65% of patients who fulfil Epstein criteria of AS (clinical stage T1c, PSA level ≤ 10 ng/mL, GS ≤ 6, PSA-D ≤ 0.15 ng/mL, one or two positive biopsy cores and percentage of core involvement ≤ 50% [24].

In our study, pathological upgrade occurred in 70.5% of patients with biopsy ISUP 1 and PIRADS 5. Similar upgrade occurred within EAU intermediate risk patients. As the risk group stratification was based on DRE and PSA results, these factors in combination with PIRADS score may predict upgrade. Currently, most often deferred treatment is excluded in patients with cT2b < / = disease and PSA above 10 ng/mL [25, 26]. Furthermore, PIRADS 5 and 4scores should be considered in deciding of radical treatment in patients with ISUP Gleason Grade Group 1.

## Limitations

We acknowledge several weak points of our study. First of all, its retrospective nature results in selection bias. Clinical stage was assessed by several physicians qualifying the patients for surgery. Therefore, these data may be subjective and heterogeneous. Prostate biopsies were performed in different institutions consequently biopsy protocols and pathological assessment are not uniform. In this group mpMRI has been performed after biopsy. It has been confirmed that use of fusion biopsy decrease the pathological upgrade on final specimen [27]. However, most patients included in our study have been treated in times when recommendation of performing mpMRI before biopsy was not strong according to EAU guidelines.

## Conclusions

This study evaluates the correlation between mpMRI, PIRADS score, and ISUP 1 PCA upgrade to significant prostate cancer on final pathology. It correlates with an upgrade on final pathology and may justify shared decision of radical treatment in patients unwilling to repeated biopsies. However, use of PIRADS 5 score as a sole indicator for prostatectomy may result in nonnegligible overtreatment rate. On the other hand, PIRADS 3 and 4 scores should be definite indication for confirmatory biopsy in the course of qualification for radical prostatectomy.

## Data Availability

The datasets used and/or analysed during the current study are available from the corresponding author on reasonable request.

## Abbreviations

PSA: Prostate specific antigen
AS: Active surveillance
DRE: Digital rectal examination
mpMRI: Multiparametric resonance imaging
PIRADS: Prostate imaging and report and data system
ISUP: International Society of Urological Pathology
EPE: Extraprostatic extension
GL: Gleason score
LPR: Laparoscopic Prostatectomy
OR: Odds ratio
CI: Confidence interval
PCA: Prostate cancer
